# Multiple Bilateral Unerupted Supplemental Premolars: An Unusual Presentation in a Nonsyndromic Patient

**DOI:** 10.5005/jp-journals-10005-1439

**Published:** 2017-06-01

**Authors:** V Satish, Suman Panda, Prabhadevi Maganur, Ather Ahmed

**Affiliations:** 1 Associate Professor, Department of Pedodontics, College of Dentistry, Jizan Kingdom of Saudi Arabia; 2 Head, Department of Pedodontics, College of Dentistry, Jizan Kingdom of Saudi Arabia; 3 Associate Professor, Department of Pedodontics, College of Dentistry, Jizan Kingdom of Saudi Arabia; 4 Assistant Professor, Department of Pedodontics, College of Dentistry, Jizan Kingdom of Saudi Arabia

**Keywords:** Bilateral premolars, Supernumerary premolars, Supernumerary teeth.

## Abstract

Supernumerary teeth are one of the routinely encountered developmental disturbances representing one or more extra teeth in the jaws. These may resemble the teeth of the group to which it belongs, i.e., molars, premolars, or incisors, or it may bear little or no resemblance. Based on the morphologic appearance, it is classified as supplemental teeth or rudimentary teeth. The supplemental teeth are those that resemble the teeth to which it is associated and rudimentary teeth are those that do not resemble the teeth. Supplemental teeth, most of the times, are associated with syndromes and they can also be present in the absence of systemic pathology. Literature reveals predilection of the presence of supplemental premolars of 2:1 in males.

This is the case report of a 14-year-old male who presented with multiple unerupted bilateral supplemental premolars on both the arches.

**How to cite this article:**

Satish V, Panda S, Maganur P, Ahmed A. Multiple Bilateral Unerupted Supplemental Premo-lars: An Unusual Presentation in a Nonsyndromic Patient. Int J Clin Pediatr Dent 2017;10(2):217-222.

## INTRODUCTION

Supernumerary teeth or hyperdontia is a mammalian developmental abnormality, which is characterized by the presence of extra teeth. This is marked by the presence of additional teeth to the regular eruption series.^1^ Around 90 to 98% of supernumerary teeth occur in the maxilla, and 90% of these are restricted to the premax-illa.^2^ Supernumerary teeth are seen in both primary and permanent dentition, but most commonly in permanent dentition; these teeth may be single or multiple, unilateral or bilateral, erupted or impacted, and in one or both jaws. Supernumerary teeth have been classified into supplemental or rudimentary. Those teeth that resemble the teeth of the group to which it belongs, i.e., molars, premolars, or incisors, are called supplemental teeth, and rudimentary teeth are those teeth that bear little or does not resemble the teeth with which it is associated. This developmental anomaly can occur either in the maxilla or in the mandible.^3^ They have frequently been seen in association with syndromes, such as Gardner’s syndrome, cleidocranial dysplasia, trichorhinophalangeal syndrome, and cleft of the lip and palate. They can also be associated in the absence of systemic pathology.^3^

This report describes a case of bilateral supplemental supernumerary mandibular premolar in a nonsyndromic patient.

## CASE REPORT

A 14-year-old male came to the dental clinic with the chief complaint of pain in the lower left back tooth region. His familial, medical, and dental histories were noncontribu-tory. The extraoral examination was noncontributory. Intraoral examination revealed a regular set of permanent dentitions. Generalized plaque and calculus accumulation were present on the teeth along with generalized gingivitis. Deep proximal caries was present with respect to 36 with tenderness on percussion.

**Fig. 1:**
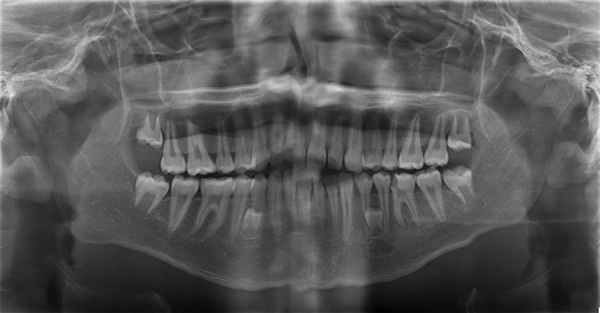
Orthopantomogram showing bilateral unerupted supplemental premolars in mandibular arch

**Figs 2A and B:**
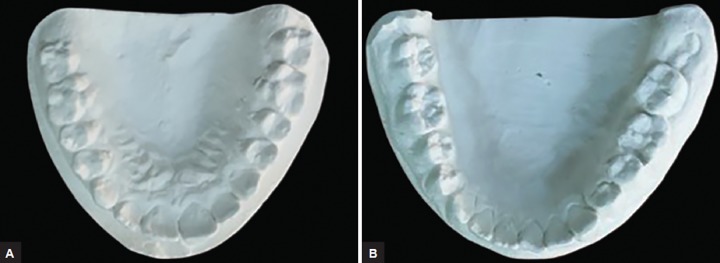
Diagnostic maxillary: (A) Mandibular; and (B) casts

An orthopantomogram (OPG) (Fig. 1) of the patient was taken to evaluate the complete dentition and the caries status. The OPG revealed two supernumerary teeth placed just between the roots of both left and right mandibular premolars. No other impacted or supernumerary teeth or any other pathologies were seen. The supernumerary teeth observed were of normal size and shape with incompletely formed roots and resembled premolars. Impressions of upper and lower arches were made, and study models were prepared (Fig. 2). The patient’s guardian was informed about the presence of extra teeth and was educated about the difficulties associated with it. As family history, medical history, general examination, and extraoral examinations were noncontributory, the diagnosis of nonsyndromic-associated supernumerary/supplemental tooth was made. Patient’s guardian was explained regarding the treatment. No intervention was planned as they were asymptomatic. Root canal treatment was initiated for 36. Patient has been kept under observation regarding the supernumerary/supplemental premolar.

## REVIEW

Excess in the total number of teeth is often termed as supernumerary teeth. The most commonly seen supernumerary teeth are^4,5^ maxillary midline supernumeraries, maxillary fourth molars, maxillary paramolars, man-dibular premolars, maxillary lateral incisors, mandibular fourth molars, and maxillary premolars.

It was found that supernumerary premolars are more common than estimated.^5^ This can be supported by the fact that 75% of these are impacted, unerupted, and asymptomatic.^4,6-8^ They are most commonly found in mandible than in the maxilla, and majority of them are of supplemental type.^4^ They are usually placed lingually or occasionally vertically below the premolar.^8,9^ According to the reports, patients who had a previous history of supernumerary teeth have a 24% possibility of developing supernumerary premolars at a later stage.^10^

## OCCURRENCE

Supernumerary tooth can occur anywhere in the dental arch, but most commonly are found in the maxilla. But supernumerary premolars are likely to occur in the mandible than in maxilla. They most often resemble the normal anatomy and morphology of premolars. It may be seen in the anterior or posterior region of the mouth, single or multiple, unilateral or bilateral, and erupted or impacted.^11^ Most of the times multiple supernumerary premolars are seen with an associated syndrome, such as Gardner’s syndrome, cleidocranial dysplasia, Fabry-Anderson syndrome, Ehlers-Danlos syndrome, Down’s syndrome, Crouzon’s disease, Hallermann-Streiff syndrome, and orodigitofacial dysostosis.^12^ But presence of multiple supernumerary premolars in the absence of any associated syndromes or systemic conditions is relatively rare.^13,14^ Multiple supernumerary teeth without any associated systemic condition or syndromes have been reviewed by Yusof.^15^ It was found that there was a predilection for nonsyndromic multiple supernumerary teeth to occur in the mandible. When analyzed according to specific locations for both maxilla and mandible, there was a predominance of multiple supernumerary teeth to occur in the premolar area (62%), with the highest frequency of occurrence in the mandibular premolar area (45%). Studies have shown an occurrence rate of 26% for supernumerary maxillary premolars and extremely high occurrence rate of 74% for supernumerary mandibular premolars.^4,15^

The supernumerary teeth have been observed in the gingiva, soft palate, nasal cavity, maxillary sinus, sphenomaxillary fissure, ophthalmic conchae, maxillary tuberosity, incisive suture, and between the orbit and the brain.^16^

## GENDER PREDILECTION

The literature suggests supernumerary tooth are most commonly seen in males than in females at a ratio of 9:2.^17^ The supernumerary premolars are more frequent in males than females. The male to female ratio has been reported as 3:1, with a mean age of 16.4 years.^5^

## INCIDENCE

The incidence of supernumerary teeth among the general population has been noticed as somewhat familiar. In southern Nigeria, 1 person in every 100 has 1 or more extra premolar teeth.^18^ Parry and Iyer^19^ reported the appearance of four supernumerary premolars in a population of 2,000 orthodontic patients, indicating incidence of 0.20%.

The incidence of supernumerary teeth in the Caucasian population as reported by Gulati and Gupta^20^ is in the range between 0.15 and 1.5%. In 1932, Stafne^4^ stated that out of 9% of all supernumeraries, 7% are mandibular and 2% are maxillary teeth.

### Prevalence

The prevalence rates of supernumerary premolars reported in different studies are varied. This is mainly due to the differences in population, age, race, and applied radiographic techniques. The prevalence range depends on the methodology for detection and variation in the population.^21^

The prevalence of supernumerary teeth varies between 0.3 and 1.9% in primary dentition and 0.1% to 3.6 to 5.3% in permanent dentition.^1^

It has been reported that the prevalence of the supernumerary premolars ranges between 0.029^22^ and 0.64%.^23^ The premolars account for only 10% of all the supernumerary cases.^4,22-24^ Single supernumeraries occur in 76 to 86% of cases, double supernumeraries occur in 12 to 23% of the cases, and multiple supernumerary teeth are very rare, accounting for less than 1% of cases.^25^

## CLASSIFICATION

Supernumerary teeth can be grouped or classified concerning chronology, topography, and morphology^13,26,27^ (Table 1).

## ETIOLOGY^12,17,27,28^

Numerous theories have been proposed for the development of supernumerary teeth (Table 2).

- Phylogenetic theory of Atavism (evolutionary throwback)
- Dichotomy theory (cleavage of a single tooth bud to two homologous or heterologous parts)
- Dental lamina hyperactivity theory.
- Combination of hereditary and environmental factors

Individuals with other dental anomalies and developmental disorders are the ones who frequently present with supernumerary teeth.

## MECHANISM OF THE SUPERNUMERARY TOOTH IN PREMOLAR REGION

Gardiner^29^ described three possible mechanisms that can give rise to a supernumerary tooth in the premolar region.

1. An abnormal proliferation of the dental lamina that can give rise to a predeciduous type of supernumerary tooth. The presence of primary supernumerary tooth indicates a significantly higher probability of a permanent supernumerary to develop because primary tooth buds generally produce an extension of the dental lamina or the formation of permanent teeth.
2. A more similar type of situation can be noted before the development of the permanent tooth takes place. The dental lamina provides an additional follicle that gives rise to a supernumerary tooth.
3. On the contrary, an additional tooth can develop from an extension of the dental lamina after the deciduous, as well as the permanent follicles have been formed. This has been termed a postpermanent type of supernumerary tooth.

**Table 1:** **Table 1:** Classification of supernumerary tooth

Chronological		Predeciduous		They develop before deciduous teeth			
		Prepermanent type		Before the development of permanent tooth		More frequent type	
		Postpermanent type		After the deciduous as well as the permanent follicles have been formed			
Morphological		Supplemental (Eumorphic) Rudimentary [conical (peg shaped), tuberculate (barrel shaped), molariform and odontome]		Those teeth that resemble the teeth of the group to which it belongs, i.e., molars, premolars, or incisors Those teeth that may bear little or no resemblance to the teeth with which it is associated are termed as rudimentary			
Topographical							
		Mesiodens		It is usually small and conical in shape. It is seen in between two maxillary central incisors		It is the most common type	
		Paramolar		It is usually small and rudimentary. It is most commonly situated buccally or palatally to one of the molars			
		Distomolar		It is a tooth located in the region posterior to the third molar tooth			

**Table 2:** **Table 2:** Etiological factors for supernumerary tooth

Theory of Atavism (Evolutionary throwback)		Oldest theory		Development of supernumerary teeth is related to phylogenetic reversion to extinct primates with three pairs of incisors		However, this theory has been discounted now	
Dichotomy theory				The splitting of the tooth bud into two equal or different-sized parts results in the formation of two teeth of equal size, or one normal and one dysmorphic tooth respectively		Accepted theory	
Dental lamina hyperactivity theory				They are found as a result of local, independent, and conditioned hyperactivity of dental lamina		Most accepted theory	
Other factors like environmental and genetic factors							

With respect to etiology of supernumerary premolars, it is evident that the dental lamina has reacted in excess to some extent. This can be justified by the fact that the location of such teeth is usually on the lingual side of the arch or vertically below the premolar teeth. This influence may be genetic since supernumerary premolars, as well as premolar hypodontia, occur frequently in the mandibular arch. It is also interesting to point out that hyperdontia has a strong predilection for males and hypodontia is more frequent in females. This finding suggests sex-linked inheritance.^30,31^

## SYMPTOMS

Usually, these teeth are asymptomatic, erupted, or impacted. Sometimes pathological sequelae are associated with it. They may be associated with cyst formation, damaging to the neighboring tooth. This happens by compression of the supernumerary premolars on the adjacent teeth, and their relation to the inferior dental and mental nerve may lead to pain. Supernumeraries can be associated with other dental anomalies, such as hypodontia, taurodontism, germination, and macrodontia.^29,32^

## DIAGNOSIS

Supernumerary teeth most of the times are asymptomatic and most of these are an incidental finding during an intraoral or radiographic examination. So, radiographic assessment is of great importance in diagnosis and management of supernumeraries. Periapical, OPG, occlusal X-rays, and advances in radiographic techniques and an introduction of three-dimensional computed tomography and cone beam supernumeraries are of paramount importance, especially in cases of multiple supernumeraries.^31^

The diagnosis is based mainly on radiographic findings. Supernumerary premolars have not become radio-graphically visible until the patient’s normal premolars have erupted.

Even though supernumerary premolars can be palpated during an intraoral examination, it should be taken into an account that supernumerary premolars can go unnoticed on intraoral X-rays when located very close to the basal cortical bone of the mandible.^23^

## ERUPTION AND COMPLICATIONS/PROBLEMS

The eruption of supernumerary premolars is very rare. Supernumeraries may show normal eruption, remain impacted or inverted in the jaw, or reach the heterotopic position or show abnormal eruptive patterns by erupting lingually or labially. In some cases, unerupted premolars can migrate distally through the mandible below the roots of the lower molars.^32,33^

### Complications/Problems^2,5,26^

It is very rare to find a supernumerary tooth in primary dentition; the problems or complications are seen mainly in the permanent dentition. The presence of supernumerary teeth may exhibit following problems:

- Malocclusion caused due to disturbance in path of eruption by reducing arch circumference and interfering with normal eruption pattern of dentition.
- Prevent eruption of developing teeth.
- External root resorption on the adjacent teeth due to pressure exerted on the erupting supernumerary teeth or resorption of adjacent structures.
- A deviated path of eruption of supernumerary teeth (nasal cavity, orbit, inferior border of the mandible, and sometimes weakening of bones to form a more prone site for fracture).
- An untreated and unerupted supernumerary tooth may transform into a dentigerous cyst.
- The supernumerary teeth may get fused with the normal teeth affecting morphology of the involved teeth, and these teeth may be impacted.
- Displacement of them (rotation and labial or lingual eruption).
- Impacted (especially primary tooth).

Supernumerary teeth can remain impacted for many years without clinical, pathologic, or orthodontic complications.

## TREATMENT

Early diagnosis and treatment permit an interceptive approach and correction of arch crowding. Management of supernumeraries depends upon the signs and symptoms as well as problems seen associated with it. The management for the supernumerary tooth can be by:

- Surgical extraction
- Monitoring
- Retaining the supernumerary premolar

### Surgical Extraction

In general terms, extraction of supernumerary premolars is the recommended treatment of choice, but the timing and surgical removal appear to be controversial. Surgical removal is recommended if it is causing any problems to permanent teeth.

Premolars are perhaps among the supernumerary teeth most difficult to extract, due to the high density of the mandibular bone, the presence of the mental nerve on the vestibular side, and the difficulties involved in approaching from the lingual side.^33^ In this context, a buccal approach is preferred, using a Neumann full-thickness flap. Raising a lingual flap is inconvenient and risky, but often necessary if the supernumerary premolar is closer to the lingual cortical and a buccal approach puts other teeth or the dental nerve at risk. When a buccal flap is used, the mental nerve must be identified where it crosses the mental foramen, to avoid postoperative sensory lesions. During ostectomy, caution is required to prevent damage to the neighboring roots and to preserve the contents of the mandibular canal. Extraction usually requires tooth section before extraction with elevators. The technique employed in the upper maxilla is similar.^33^

Marre^34^ and Hanratty^35^ recommended surgical removal in two stages: in the first stage, removal of the developed supernumerary premolars should be accomplished soon after diagnosis. In the second stage, the remaining less-developed premolars are left *in situ* and removed later, when their roots are more developed, to avoid damage to adjacent structures and allow bone regeneration.

### Monitor

It has been recommended by Becker et al^36^ that supernumerary premolars be left untreated until the full permanent dentition has developed and erupted. Because supernumerary premolars develop late with respect to the erupted teeth, the treatment of the overall condition may be referred and should involve orthodontic treatment of erupted as well as unerupted teeth. Routine follow-up instead of extraction is also a possibility, though it requires a periodic radiographic evaluation of the tooth to detect possible cyst formation or root resorption. After formation of the third molars, supernumerary premolar extraction may be indicated; in this sense, care is required to ascertain that no other supernumerary premolars can arise following extraction of those that have been identified, as this would require repeated surgery.^33^ Removal involves risk to adjacent structures.

### Retain the Supernumerary Tooth

Supernumerary teeth can be utilized in cases of congeni-tally missing or teeth extracted due to other reasons for replacing the function of that particular tooth by proper restoration.

## CONCLUSION

- Supernumerary premolars occur more commonly in males when compared with females.
- Supernumerary premolars are more associated with mandible than the maxilla.
- Radiographs are very important for diagnosis and management of supernumerary premolars.
- Treatment of supernumerary premolars depends on whether they are symptomatic or asymptomatic.
